# Enteric Methane Emissions Prediction in Dairy Cattle and Effects of Monensin on Methane Emissions: A Meta-Analysis

**DOI:** 10.3390/ani13081392

**Published:** 2023-04-18

**Authors:** Joyce L. Marumo, P. Andrew LaPierre, Michael E. Van Amburgh

**Affiliations:** Department of Animal Science, Cornell University, Ithaca, NY 14853, USA

**Keywords:** monensin, dairy cattle, enteric methane emissions, methane production, methane prediction equation, dry matter intake, empirical modeling

## Abstract

**Simple Summary:**

Enteric methane (CH_4_) emissions are a global concern and have been associated with climate change. Thus, sustainable, easily applicable CH_4_ mitigation strategies should be in place without having an adverse effect on animal productivity. We (i) developed a series of dairy cattle enteric CH_4_ production (g/d) and yield (g/kg of dry matter intake, DMI) models using combined (lactating and non-lactating cows) and lactating data, (ii) investigated the effects of monensin on enteric CH_4_ emissions in dairy cattle, and (iii) evaluated the proposed and published models. Monensin reduced daily CH_4_ production and CH_4_ yield by 5.4% and 4.0%, respectively. Further, long-term in vivo studies on monensin feeding of ≤24 mg/kg DM with CH_4_ measurements taken to account for bacterial adaptation in the rumen are needed. Overall, DMI is the significant driver of CH_4_ emissions in dairy cattle and a model that included DMI, dietary forage proportion, and the quadratic term of dietary forage proportion was the best model for both combined (lactating and non-lactating) and lactating cows. The methane yield was best predicted with dietary forage only for combined data, while a combination of dietary forage proportion, milk fat, and milk protein yields was the best model for lactating cows. This indicates that the inclusion of dietary composition along with DMI can provide a better CH_4_ production prediction in dairy cattle. The selected developed models outperformed the published models.

**Abstract:**

Greenhouse gas emissions, such as enteric methane (CH_4_) from ruminant livestock, have been linked to global warming. Thus, easily applicable CH_4_ management strategies, including the inclusion of dietary additives, should be in place. The objectives of the current study were to: (i) compile a database of animal records that supplemented monensin and investigate the effect of monensin on CH_4_ emissions; (ii) identify the principal dietary, animal, and lactation performance input variables that predict enteric CH_4_ production (g/d) and yield (g/kg of dry matter intake DMI); (iii) develop empirical models that predict CH_4_ production and yield in dairy cattle; and (iv) evaluate the newly developed models and published models in the literature. A significant reduction in CH_4_ production and yield of 5.4% and 4.0%, respectively, was found with a monensin supplementation of ≤24 mg/kg DM. However, no robust models were developed from the monensin database because of inadequate observations under the current paper’s inclusion/exclusion criteria. Thus, further long-term in vivo studies of monensin supplementation at ≤24 mg/kg DMI in dairy cattle on CH_4_ emissions specifically beyond 21 days of feeding are reported to ensure the monensin effects on the enteric CH_4_ are needed. In order to explore CH_4_ predictions independent of monensin, additional studies were added to the database. Subsequently, dairy cattle CH_4_ production prediction models were developed using a database generated from 18 in vivo studies, which included 61 treatment means from the combined data of lactating and non-lactating cows (COM) with a subset of 48 treatment means for lactating cows (LAC database). A leave-one-out cross-validation of the derived models showed that a DMI-only predictor model had a similar root mean square prediction error as a percentage of the mean observed value (RMSPE, %) on the COM and LAC database of 14.7 and 14.1%, respectively, and it was the key predictor of CH_4_ production. All databases observed an improvement in prediction abilities in CH_4_ production with DMI in the models along with dietary forage proportion inclusion and the quadratic term of dietary forage proportion. For the COM database, the CH_4_ yield was best predicted by the dietary forage proportion only, while the LAC database was for dietary forage proportion, milk fat, and protein yields. The best newly developed models showed improved predictions of CH_4_ emission compared to other published equations. Our results indicate that the inclusion of dietary composition along with DMI can provide an improved CH_4_ production prediction in dairy cattle.

## 1. Introduction

The abatement of greenhouse gases (GHG), mainly methane (CH_4_), and its environmental effects on climate change is a global concern. Despite CH_4_ having a short lifetime in the atmosphere with an average of 12.4 years [1], it is 28–34 times more potent than carbon dioxide at causing global warming over a century [2] with a significant impact on climate change. Ruminant enteric CH_4_ gas is an end-product of the rumen fermentation process which is influenced by dietary components and is responsible for 2–12% of overall feed energy loss [3,4] and is diverted away from animal productivity. Within the livestock sector, small ruminants and buffalo are responsible for 15.4% of the sector’s CH_4_ emissions, while dairy and beef cattle account for the majority of these emissions (30% and 35%, respectively) [5]. To counter these CH_4_ emissions, over 100 countries launched a Global CH_4_ Pledge in November 2021, agreeing to reduce CH_4_ by 30% from 2020 levels by the year 2050 [6].

Enteric CH_4_ mitigation strategies in ruminants include but are not limited to, dietary feed formulation changes [7], the use of feed additives/rumen modifiers such as ionophores [8], essential oils [9], plants extracts [10], plant secondary metabolites (e.g., tannins) [11], and chemical inhibitors [12] along with genetics and management [13]. All these strategies have been shown to reduce enteric CH_4_ production either directly or indirectly; however, consistent and cost-effective strategies are not yet established [2].

The quantification of enteric CH_4_ emissions using in vivo CH_4_ measurement techniques can be costly; therefore, an effort in the development of empirical models has been made and continues to increase [14,15,16,17,18,19,20]. Other empirical models have demonstrated that including diet composition variables make a significant contribution to enteric CH_4_ production accurately [16,18,21]. An effort was taken by IPCC [19] in the development of empirical models for a wide range of animals; however, several studies have observed an inaccuracy in enteric CH_4_ production values with the use of this model when evaluated on an intercontinental database comprised of Europe, North America, Australia, Asia, and South America data [16,22]. Thus, nutritionists must understand the impact of dietary changes and their influence on CH_4_ emissions. The incorporation of CH_4_ prediction models in diet formulation models is vital as that will assist in the decision-making process to enhance animal productivity while reducing its environmental impact.

The use of non-nutritive supplements (feed additives) such as monensin has proven to improve CH_4_ predictions in the VFA stoichiometry [23,24] despite the fact that a variety of empirical models have demonstrated that dietary nutritional composition plays a significant role in ruminant enteric CH_4_ production [16,18,21]. A meta-analysis was published which attempted to describe monensin’s effect on CH_4_ emissions in dairy and beef cattle [25]; however, to our knowledge, no study has developed empirical predictive CH_4_ emission models exclusively on the use of monensin as a Food-and-Drug-Administration-approved feed additive with CH_4_ mitigating properties. The use of cost-effective CH_4_ quantification strategies, such as the use of empirical predictive models developed from particular feed additives, would be useful as they will provide an estimation of the reduction in CH_4_ production without the need to acquire expensive equipment.

The ionophore monensin is frequently used in cattle diets to increase feed energy utilization efficiency [26]. It can reduce enteric CH_4_ formation by reducing methanogens, which favors propionate production, which then utilizes a methyl group for the additional carbon to dispose of hydrogen [27]. 

However, there are inconsistencies in the effectiveness of ionophore feeding in cattle methanogenesis. For instance, Van Vugt et al. [28], O’kelly and Spiers [29], Odongo et al. [30] observed a significant reduction of over 6.0 % in the daily enteric CH_4_ production of over a 50 d feeding period, whereas others did not [9,31,32,33,34]. The recent National Academy of Sciences, Engineering, and Medicine, Nutrient Requirement of Dairy Cattle (NASEM) [35] stated a 5% reduction in CH_4_ emissions with the use of monensin in diet formulation. In addition, previous studies reported a temporary decrease in CH_4_ production [36,37,38], stating that the ruminal microbial population adapted to supplementation, but no evidence of supplementation impact on methanogens was directly investigated.

Earlier research has shown that monensin efficacy in reducing methanogenesis is dependent on its dosage [27], dietary forage content, as well as feeding duration [24,36,39]. For example, Odongo et al. [30], Mbanzamihigo et al. [40], Davies et al. [41] observed a significant decrease in CH_4_ production after long-term feeding without evidence of ruminal microbial adaptation (40–240 days). Furthermore, De and Singh [42] found a minimum adaptation period of 21 days for monensin on microbial fermentation/cell wall digestibility and CH_4_ production reduction, and this is consistent with the effect of monensin on milk fat composition where changes were reversed after an 18 d washout period [43]. 

The objectives of this paper were to: (i) develop a dairy cattle database to accurately predict the enteric CH_4_ production of animal records and investigate the inhibitory effects of monensin supplementation on CH_4_ emissions; (ii) identify the key predictor variables for predicting dairy cattle enteric CH_4_ production (g/d) and yield (g/kg DMI); (iii) develop dairy cattle enteric CH_4_ emissions prediction models; and (iv) evaluate the proposed models and compare these results to the previously published models.

## 2. Materials and Methods

### 2.1. Model Database Construction

#### Monensin Database

Literature search. A literature search was conducted using Scopus and Web of Science databases, whereby an initial search resulted in a total of 333 published research papers from 1981 to 2020. For the search of the literature, a combination of terms was used: “monensin”, “methane”, and “cattle”, or “cow”, or “ruminant”.

Eligibility criteria. For the studies to be included in the database, the following eligibility criteria were established: (i) the studies should be in vivo and involve monensin feeding using dairy cattle; (ii) the studies should have a control group that did not receive monensin; (iii) the studies reported CH_4_ production as the response/outcome for both the control and a monensin treatment group; and (iv) the studies reported the treatment means of other variables such the observed dry matter intake (DMI), dietary composition, and lactation performance for studies that included lactating cows.

Selection process. Following the removal of duplicates and null entries (80 papers), 253 papers were identified. Then, a thorough screening process of records to identify the potential eligible papers based on the eligibility criteria resulted in the elimination of 135 papers from the database (in vitro papers (100), paper in another language (1), meta-analyses (5), review papers (22), simulation papers (3), editorial and erratum papers (2), papers in goats and sheep (2)). This resulted in a total of 118 papers, and we removed an additional 77 papers that did not fulfill the criteria. The search resulted in 41 papers that were related to monensin feeding effects on CH_4_ emissions; therefore, a further reading of full-text articles of the title, abstracts, experimental designs, and results was conducted. Out of 41 papers, 17 articles were excluded for the following reasons: summary papers (4), no CH_4_ emission measurements (2), no monensin/treatment effect results reported (2), without control measurements (3), conference papers as there were duplicate publications of the similar study (2), conference paper only reported an abstract, simulation papers (2), and paper that investigated the effects of monensin when mixed with other feed additives. The final database had a total of 24 papers that met the criteria and remained in the database; however, 13 were related to beef cattle [29,36,38,39,44,45,46,47,48,49,50,51,52] and 11 were related to dairy cattle [8,9,28,30,31,32,33,34,53,54,55]. Then, beef cattle papers were excluded from the database. Finally, only the dairy cattle database was considered for the present study, except those conducted under grazing conditions, i.e., four studies (*n* = 8 treatment means) Van Vugt et al. [28]; two studies (*n* = 4 treatment means; Grainger et al. [34]); and two studies (*n* = 4 treatment means; Grainger et al. [33]). Figure S1 illustrates a flowchart of the data searching, screening, and selection process used in the current study. For the investigation of the effects of monensin supplementation on CH_4_ emissions using a monensin database, only studies that had taken CH_4_ measurements ≥21 d following monensin feeding were considered. Regardless of the delivery method (i.e., controlled-release capsule (CRC) or premix) and CH_4_ measurement technique (i.e., respiration chamber, SF_6_, hood) utilized to measure enteric CH_4_ monensin’s CH_4_, the inhibitory effects were the same.

The database was assessed for outliers using the interquartile range (IQR) as described by Kokoska and Zwillinger [56], whereby a factor of 1.5 was regarded as extreme. The outliers were assessed using the CH_4_ production, CH_4_ yield (g/kg of DMI), and DMI. The study of Hamilton et al. [54] reported an unrealistic low CH_4_ production with both the control and monensin treatment containing 35% forage and DMI > 28 kg/day (103 ± 37 days in milk); therefore, the values were considered outliers and removed from the database for the final analysis. 

To achieve the third objective, additional papers were added to the database for equation development, and the literature search was conducted through the same databases. Moreover, grazing studies were not considered in the database because most papers did not report the actual DMI. The paper was included in the database if it had met the following inclusion criteria: (i) had reported DMI, dietary components including neutral detergent fiber (NDF), acid detergent fiber (ADF), lignin/acid detergent lignin (ADL) except in two studies, and/or hemicellulose/cellulose; (ii) was written in English; (iii) was carried out as an in vivo study; and (iv) did not investigate any other feed additives and measured CH_4_ production as an outcome. In the case where hemicellulose and cellulose were not reported, they were calculated as described below. In two studies, the lignin content was not reported; therefore, values from the Cornell Net Carbohydrate and Protein System (CNCPS) feed library [57] were procured and matched based on the feeds which most closely aligned with those fed in the study.

The final dataset comprised 61 observations from 18 studies (combined, COM database) including lactating (*n* = 48 treatment means; LAC) and non-lactating dairy cattle (*n* = 13 treatment means; NLAC). The non-lactating cows included heifers (*n* = 4) and dry cows (*n* = 9). The COM database included both monensin and additional papers. The summary of the studies used in the current database is shown in Table 1.

### 2.2. Data Extraction and Calculations

Animal, dietary, and lactation performance factors were all considered in the developed databases. A preliminary analysis demonstrated that DMI along with dietary variables such as lignin as a percentage of NDF, (Lig.%NDF), cellulose, and hemicellulose/cellulose (H:C) ratio were the significant predictors of enteric CH_4_ production. Thus, the parameters used included DMI (kg/d), intake energy (IE, MJ/d), metabolizable energy intake (MEI, MJ/d), and dietary nutrient composition (crude protein, CP; NDF; ADF; Lig.%NDF, acid detergent lignin, ADL; ether extract, EE; ash; hemicellulose, cellulose, H:C ratio, non-fiber carbohydrates, NFC; and forage proportion (all expressed as % of DM)). Lactation performance factors included milk yield (MY, kg/d); milk fat yield (MFY, g/d); milk protein yield (MPY, g/d); and energy-corrected milk (ECM, kg/d, Tyrrell and Reid [70]). Furthermore, for the development of the monensin database, other input variables such as the monensin dose (mg/kg DM of feed), monensin delivery method (CRC or premix), duration of monensin feeding (days), and the number of days of CH_4_ production measurements after monensin feeding were considered in the database and the number of animals per study were associated with each treatment mean.

For studies with missing nutrient composition variables, the nutrient composition for individual feed ingredients were populated using the feeds selected from the CNCPS feed library [57] provided the studies had reported adequate dietary ingredient descriptions. Additionally, in most cases, there was no hemicellulose and cellulose reported, so hemicellulose was calculated as NDF—ADF, while cellulose was calculated as ADF—ADL [71]. Moreover, other missing variables, IE, NFC, MEI, and ECM, were obtained using the following equations: ECM was calculated as ECM (kg/d) = 12.95 × milk fat yield (kg/d) + 7.65 × milk protein yield (kg/d) + 0.327 × milk yield (kg/d) Tyrrell and Reid [70]; NFC was calculated as NFC (%) = 100 − (NDF + CP + EE + Ash), where all nutrient composition variables were expressed as % of dietary DM. When IE was not reported, it was estimated from the DMI and dietary nutrient composition as cited by Ramin and Huhtanen [72] IE (MJ/d DM) = DMI (kg/d) × [(23.6 × CP + 39.8 × EE + 17.3 × NFC + 18.9 × NDF)/100] and the CH_4_ conversion factor (Y_m_, %) was calculated as CH_4_ production (g/d) × 0.05565 ÷ IE (MJ/d) × 100). The estimation of MEI was calculated first from TDN using the equation TDN (%) = 92.2 − 1.12 × ADF [73], whereby 1 kg TDN = 4.409 Mcal/kg DE [74]. Then, to calculate ME: ME (Mcal/kg) = −0.45 + 1.01 × DE (Mcal/kg) [74]. Finally, MEI was calculated as the ME content multiplied by the DMI associated with each treatment mean. 

In the studies where the CH_4_ yield and intensity were not reported, the CH_4_ yield values were calculated by dividing CH_4_ production by the measured DMI (kg/d) and CH_4_ intensity by dividing the CH_4_ production by the provided ECM (kg/d) or MY (kg/d). In the current database, the majority of the studies expressed CH_4_ production in g/d, thus, in cases where it was reported in other units such as MJ/d or L/d, it was then converted as follows: 22.4 L CH_4_ = 16.0 g (1 L CH_4_ = 0.716 g CH_4_) and 1 g CH_4_ = 55.6 KJ (0.0556 MJ).

### 2.3. Statistical Analyses

All data analyses were carried out using R Statistical language (version 4.1.2 (1 November 2021, R Foundation for Statistical Computing, Vienna, Austria)) in RStudio version 2022.7.1.554 [75]. The data were analyzed using a linear mixed model fitted with lmer (lme4 package) [76] using this model:

(1) $$\;Y=\beta_{0}+\beta_{1\;}X_{1}+\beta_{2}X_{2}+\;\ldots \ldots +\beta_{n}X_{n}+S_{i}+e_{ij}$$

where *Y* denotes the expected outcome of the dependent variables of CH_4_ production (g/d), CH_4_ yield (g/kg DMI), or CH_4_ intensity (g/kg ECM). $\beta_{0}$ denotes the fixed effect of the random intercept, *X*_1_ to *X*_n_ denote the fixed effects of the independent variables, *β*_1_ to *β*_n_ denote their corresponding slopes, *S_i_* denotes the random effect of the studies, and *e_ij_* denotes the random error.

#### 2.3.1. Effects of Monensin on CH_4_ Emissions

To investigate the inhibitory effects of monensin on CH_4_ emissions, only the studies that had taken measurements ≥21 days after monensin feeding were considered in the analyses. As a result, a total of 3 studies (*n* = 6) were retained in this dataset. 

#### 2.3.2. Model Variable Selection and Model Development

The CH_4_ emission regression equations were developed and evaluated on the COM and subset LAC datasets. Furthermore, in order to investigate the lactation performance variables (MY, MFY, MPY, and ECM) on CH_4_ emissions, additional categories of CH_4_ production and yield models were developed using only the LAC dataset (*n* = 48).

Simple and multiple linear mixed models to predict CH_4_ emissions were developed with data weighted by the number of animals associated with each treatment mean in order to account for the accuracy of the reported treatment means using the WEIGHT statement in the lmer function [77]. In the overall dataset, CH_4_ production was quantified using respiration chamber data in 11 studies, hood calorimetry in 2 studies, and SF_6_ in 5 studies. Data from Hammond et al. [78] observed a lack of concordance between the CH_4_ emission measurement techniques (respiration chamber, SF_6,_ and GreenFeed System, C-Lock, Inc., Rapid City, SD, USA); accordingly, prior to the final development of the model, the effect of CH_4_ production measurement techniques on the CH_4_ emissions (enteric CH_4_ production and yield) was assessed but was not significant (*p* > 0.05), thus they were excluded in the model developments as fixed effects.

For the COM database, seven CH_4_ production model categories were developed that included DMI only (DMI_S), IE only (IE_S), MEI only (MEI_S), DMI and dietary forage proportion (DMI_For_M), DMI, dietary forage proportion and quadratic term of dietary forage proportion (DMI_For_nl), DMI and dietary components variables (DMI_diet_M), and DMI and other dietary composition variables except for dietary forage proportion (noForage_diet_M). Model variable selection for the DMI_diet_M and noForage_diet_M was done using the COM data and evaluated the same retained variables on LAC data.

For the LAC data, the lactation performance variables in addition to animal and dietary input variables on CH_4_ emissions were incorporated into the models, whereby an additional seven CH_4_ production model categories were developed from one or more predictor variables that included: MY only (MY_S), ECM only (ECM_S), DMI and NDF (DMI_NDF_M), DMI and ADF (DMI_ADF_M), DMI, dietary components, and lactation performance variables (DMI_diet_lac_M), all dietary composition except DMI (noDMI_diet_M), and NDFd, dietary components, and lactation performance variables (NDFd_diet_lac_M). The DMI was used to calculate the CH_4_ yield; therefore, it was not used in the development of all CH_4_ yield models [16]. The CH_4_ model categories were: NDF only (NDF_S), ADF_only (ADF_S), dietary forage proportion only (Forage_S), dietary composition (diet_M), and animal, dietary composition, and lactation performance variables (diet_lac_M). Model variable selection for the diet_lac_M model category was performed using LAC data only.

Factors that predicted CH_4_ production were chosen using the backward elimination approach using the Akaike information criterion corrected for the sample size (AICc) in the MuMIn package [79] in models with an increased complexity (i.e., more variables); then, the model with the lowest AICc value was selected. With a small sample size, the AICc function prevents model overfitting or complexity [80,81].

Multicollinearity amongst the variables was assessed using the variance inflation factor (VIF) with a threshold of 5. A VIF greater than 5 was regarded as a signal of multicollinearity; therefore, the variable with the largest VIF was removed then the model was refit and evaluated again. To ensure the stability of the coefficients in the models, all the retained input variables in the newly developed models were significant at *p* ≤ 0.05 [82]. In order to maximize the use of the number of observations for model development, due to the limited number of observations for NDF digestibility (NDFd), the model selection was performed with the reduced dataset (COM: *n* = 43; LAC: *n* = 30) and if the model selection excluded NDFd, then the final model was refitted with the full dataset.

In the case where slope biases were found in the proposed and published models, biases were assessed using the residuals and the predicted values (mean-centered) as described by St-Pierre [83]. The models’ residual diagnostics and influential observations were also assessed [84].

#### 2.3.3. Proposed Models’ Evaluation and Cross-Validation

The leave-one-out cross-validation procedure (LOOCV) was used to evaluate the predictive ability of the developed proposed models, whereby studies were regarded as the folds. In each iteration, one study was used as the validation and the remaining studies were used as the training dataset [85]. The models developed were evaluated on COM (*n* = 61) and LAC (*n* = 48) data. The model performance metrics were calculated from the model predictions generated from the cross-validation process and published CH_4_ emissions models were also evaluated in the current study [16,17,18,19,20,72,86,87,88,89,90,91,92] on COM and LAC data only. These published models were selected based on the availability of the input variables in our database and are commonly used to predict CH_4_ production. The Nielsen et al. [91] model utilized dietary digestible NDF (dNDF, % of DM) and fatty acids (FAs) contents, which were not reported in some cases in our databases. Therefore, dNDF was then calculated as dNDF (% of DM) = [(NDFd × NDF (% of DM))/100], while FAs were replaced with EE instead. Lin’s concordance correlation coefficient (CCC) was calculated using the epiR package [93]. The CCC is the product of precision (*r*) or Pearson’s correlation coefficient and accuracy or bias correction factor (*C_b_*); a greater coefficient is an indication of a better model performance. The *C_b_* specifies how far the regression line deviates from a line at 45 degrees, and the value closer to 1 implies a better fit.

The total mean square prediction (MSPE) was calculated following the recommendation of Bibby and Toutenburg [94] using the equation below to identify systematic biases. The total MSPE was decomposed into mean bias (MB) and slope bias (SB). Both the MB and SB were expressed as the percentage of the MSPE. The root mean squared prediction error (RMSPE) was calculated and expressed as the fraction of the observed mean (expressed in g/d or g/kg DMI), and a smaller value indicates a better overall model predictive ability. To evaluate the model predictive ability given the data variability, the RMSPE-observations SD ratio (RSR) was also calculated as the RMPSE divided by the observed standard deviations.

(2) $$MSPE=\frac{\sum_{i=1}^{n}{\left(y_{i}-{\hat{y}}_{i}\right)}^{2}}{n}$$

where *y_i_* represents the observed value of the response variable for the *i*th observation and *ŷ_i_* represents the predicted value of the response variable for the *i*th observation.

(3) $$MB={\left(\bar{P}-\bar{O}\right)}^{2}$$

(4) $$SB=\left(Sp-r\times So\right)2$$

where $\bar{P}$ and $\bar{O}$ represent the predicted and observed means, respectively, *Sp* and *So* represent predicted and observed standard deviations, respectively, and *r* represents the Pearson correlation coefficient. The best models were selected based on the lowest RMSPE and highest CCC values.

## 3. Results

### 3.1. Database Description

The COM and LAC datasets description for the dietary composition and lactation performance variables and their summary statistics are shown in Table 2. On average, DMI, IE, and MEI were greater for the lactating cows, but a greater variability was found in both lactating and non-lactating cows than in LAC data. The COM data were mostly comprised of lactating cows (LAC database; 78.7%, *n* = 48 from 14 studies) than non-lactating cows (NLAC database; 21.3%, *n* = 13 from 4 studies). The range of the forage proportion in the experimental diets fed to both lactating (LAC) and combined (COM) cows was similar (40 to 100%). In the COM and LAC databases, 43 of 61 (12 studies; 70.5%) and 30 of 48 treatment means (8 studies; 62.5%) had reported NDFd. The mean NDFd was slightly greater in the combined cows (COM) than in lactating cows (LAC) (48.8 vs. 47.7%). Most of the studies used respiration chambers to measure CH_4_ production (61.1%, *n* = 44); five studies utilized SF_6_ (27.8%, *n* = 9), and two studies used hood calorimetry (11.1%, *n* = 6). In the entire database, the total dietary IE lost as CH_4_ (Y_m_) ranged from 3.2 to 7.9% of gross energy. 

In the LAC subset, MY, MFY, and MPY ranged from 10.0 to 46.3 kg/d, 490 to 1780 g/d, and 370 to 1430 g/d, respectively. On average, LAC emitted a greater daily CH_4_ production of 389.7 g/d (*SD* ± 102.68) than combined cows (346.4 ± 127.13 g/d). However, it was more variable in combined cows ranging from 147.4 to 631.2 g/d. The methane yield (g/kg DMI) was much more variable (ranging from 13.5 to 27.0) in combined cows than in lactating cows (15.2 to 25.9). On average, the CH_4_ intensities, expressed as both g/kg MY and g/kg ECM, were 16.1 (*SD* ± 2.83) and 14.97 (*SD* ± 1.97), respectively. The CH_4_ conversion factor (CH_4_, % of IE) of all cows was slightly greater (6.2 vs. 6.0 %) than that of lactating cows. There was no difference observed in the CH_4_ production between the CH_4_ collection techniques (*p* > 0.05).

### 3.2. Effects of Monensin on CH_4_ Emissions

We were unable to construct robust dairy cattle enteric CH_4_ production prediction models because the monensin database had too few observations overall (*n* = 14 treatment means). However, we were able to investigate the effects of monensin on CH_4_ emission considering studies (*n* = 6 treatment means from 3 studies) that had taken CH_4_ measurements ≥ 21 days following monensin feeding. 

We found a significant decrease in CH_4_ production at 24 mg/kg DM monensin in the diet (*β* = −25.33 ± 2.91, *t* = −8.72, *F* = 76.00, *p* = 0.013) compared to the control treatment, with the control emitting higher CH_4_ production (g/d) (*M* = 480.9, *SEM* = 12.8) than the monensin-supplemented group (*M* = 455.6, *SEM* = 12.8). Similarly, a significant decrease in the average CH_4_ yield (g/kg DMI) was found in the monensin treatment group (*β* = −0.87 ± 0.176, *t* = −4.91, *F* = 24.14, *p* = 0.04) compared to the control group. A greater CH_4_ yield was found in the control group (*M* = 22.2, *SEM* = 0.66) than the monensin group (*M* = 21.4, *SEM* = 0.66). However, no significant difference in CH_4_ intensity (g/kg ECM) was found between the monensin and control group (*F* = 1.24, *p* = 0.22).

### 3.3. Methane Production (g/d) and CH4 Yield (g/kg DMI) Models

#### 3.3.1. Methane Production Equations

**Figure 1:**
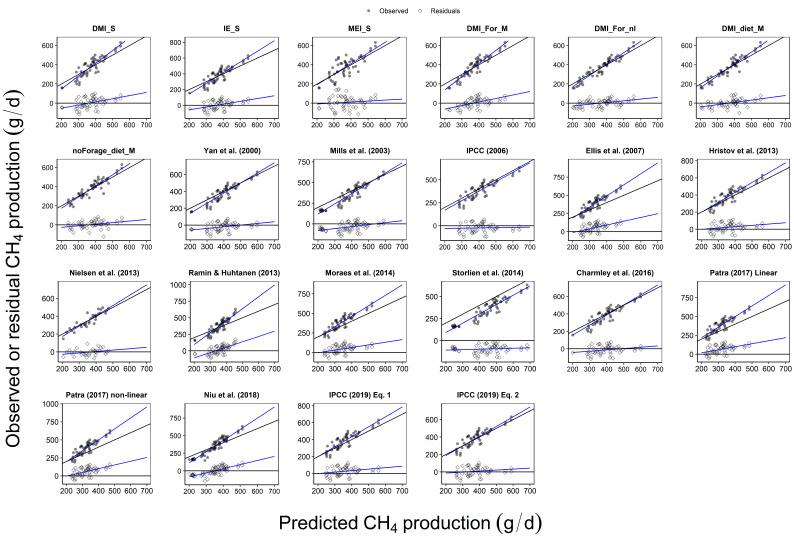
Plots of observed vs. predicted methane (CH_4_) production (g/d) (illustrated by circles), and residuals (diamond: observed—predicted values) vs. predicted methane production (g/d) (illustrated by diamond shapes) generated from the combined (COM) database (*n* = 61) from different model categories and extant equations presented in accordance with Table 3 and Table 4. The references interpretations are stated in Table 4. The solid blue lines indicate the relationship between predicted and observed methane production and predicted values and the residuals. The solid black lines represent the line of unity, where y = x (1:1) [16,17,18,19,20,72,86,87,88,89,90,91,92].

**Figure 2:**
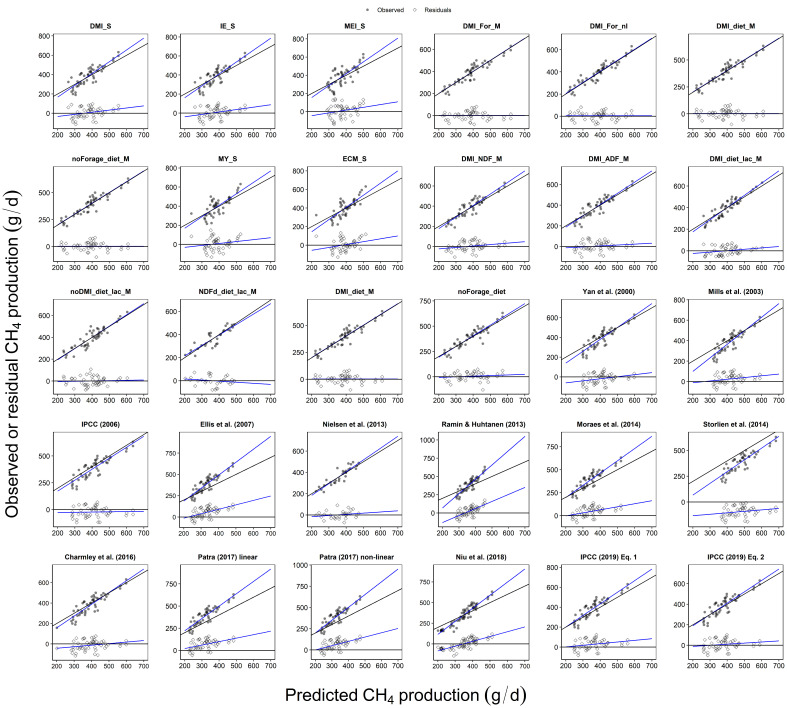
Plots of observed vs. predicted methane (CH_4_) production (g/d) (illustrated by circles), and residuals (diamonds: observed—predicted values) vs. predicted methane production (g/d) (illustrated by diamond shapes) generated from the lactating cows (LAC) database (*n* = 48) from different model categories and extant equations presented in accordance with Table 3 and Table 4. The references interpretations are stated in Table 4. The solid blue lines indicate the relationship between predicted and observed methane production, and predicted values and the residuals. The solid black lines represent the line of unity, where y = x (1:1) [16,17,18,19,72,86,87,88,89,90,91,92].

The newly developed CH_4_ production (g/d) equations, as well as the models published in the literature and their metrics, are in Table 3 and Table 4, respectively (illustrated in Figure 1 and Figure 2). The RSR values were used to evaluate the model’s predictive ability given the data variability [95]. The lactation performance and dietary variables were included in the model development using the LAC database, but for the COM database, only DMI, IE, MEI, and dietary composition variables were considered. Model variable selection in DMI_diet_M (Eqs. 6, and 13) and noForage_diet_M (Eqs. 7, and 14) was performed with COM data and we evaluated the models also using the LAC database. For the CH_4_ production regression equation development using both COM and LAC databases, collinearity was found between DMI and IE (COM: *r* = 0.80, *p* < 0.001; LAC: *r* = 0.99, *p* < 0.001) and DMI and MEI (COM: *r* = 0.97, *p* < 0.001; LAC: *r* = 0.94, *p* < 0.001); therefore, the models were developed only with DMI due to its effectiveness in predicting CH_4_ production and ease of application on the farm. Moreover, this was also supported by the lowest RMSPE values (Table 3), and models with DMI showed a better prediction compared to the models fitted with either IE or MEI (results not shown).

As expected, DMI (Eqs. 1, 4–8, 11–12, 14–16, 18–19, and 21), IE (Eqs. 2, and 9), MEI (Eqs. 3, and 10), forage proportion (Eqs. 4–6, and 11–13), MY (Eq. 15), ECM (Eq. 16), MFY (Eqs. 18, and 19), NDF (Eq. 17), ADF (Eq. 18), cellulose (Eq. 20), and NDFd (Eq. 21) were positively correlated with CH_4_ production, while Lig.%NDF (expressed as lignin as a % of NDF, Eqs. 6–7, and 13–14), H:C ratio (Eqs. 7, and 14), MPY (Eqs. 19), and the EE (Eq. 20) showed a negative relationship to CH_4_ production with both the COM and LAC databases (Table 3).

Models with DMI as the only predictor variable (Eqs. 1, and 8) did not perform better compared to multiple regression equations 4–7, 11–14, 17–19, and 21 (Table 3) based on COM and LAC databases with the highest RMPSE (>14.0%) and lowest CCC (<0.90). However, this DMI_S model did not demonstrate a low mean bias (MB = 0.07 to 1.69%) with both COM and LAC databases but showed a high slope bias on COM (SB = 17.6%). The DMI simple regression model (DMI_S) on COM and LAC databases outperformed the IE_S model (Eqs. 2, and 9: highest RMPSE = 15.1 and 14.5%; and lowest CCC value = 0.89 and 0.79, respectively) and the MEI-only model (Eqs. 3, and 10: highest RMPSE = 17.8 and 18.3%; lowest CCC value = 0.86 and 0.63, respectively). An increase in model complexity by the addition of dietary variables to the model with DMI (DMI_S: Eqs. 1, and 8) showed an improvement in the prediction performance for both databases. For the COM database, based on the lowest RMSPE and highest CCC values, the overall best model included DMI, dietary forage proportion, and the quadratic term of dietary forage proportion (DMI_For_nl (Eq. 5): RMSPE = 10.7%; CCC = 0.95) and the second best-ranked model included DMI, Lig.%NDF, and H:C ratio (noForage_diet_M (Eq. 7): RMSPE = 10.9%; CCC = 0.95), with both models revealing no systematic biases (Figure 1). The noForage_diet_M (Eq. 7) is the model that resulted when DMI and all other dietary variables were included except forage proportion, and it was first generated with the reduced dataset (*n* = 43) with NDFd included to maximize the use of the data. However, the NDFd fell out of the model selection, and the model was refitted with the full dataset. 

For the LAC database, simple regression models of milk yield (MY_S; Eq.15) and ECM (ECM_S; Eq. 16) had a similar predictive ability with RMSPEs of 16.0 and 15.7%, respectively, and greater RSR (≥0.65), with the tendency to underpredict at the upper end of CH_4_ production, and they had a lower precision (or accuracy) than Eq. 8 (DMI_S, Table 3). However, an improvement in the model performance was observed when DMI, MFY, and MPY were retained in the DMI_diet_lac_M model (Eq. 19), and this is supported by the lowest RMSPE and greater CCC values (9.8% and 0.90, respectively) (Figure 2).

In contrast to Eq. 19 (DMI_diet_lac_M), a slight increase in the prediction error of 10.1% was observed when DMI was excluded in the model development (in Eq. 20; noDMI_diet_lac_M). Overall, based on the lowest RMSPE (9.8%) and highest CCC (0.90) values, Eq. 19 (DMI_diet_lac_M) with 93.3% error to random variation demonstrated a better precision than other models generated on the LAC database (Table 3) with no system biases. This model was outperformed by Eq. 12, which parameterized the DMI, dietary forage proportion, and quadratic term of dietary forage proportion, and was also supported by the highest CCC = 0.94. For the LAC database, Equation 12 based on DMI and dietary components, and Eq. 19 based on DMI, dietary components, and lactation performance variables, outperformed all the newly developed models from this database.

Although the model variable selection in Table 3 was developed with COM data for DMI_diet_M (Eq. 6) and noForage_diet_M (Eq. 7) models, these models showed a better prediction accuracy on the LAC database (Eqs. 13, and 14) with no evidence of systematic biases.

Published model evaluation. All extant models were selected based on the availability of the input/predictor variables in our database, their frequency of use, and some of the recommendations provided by NASEM [35]. The performance of the published models on the COM and LAC databases is demonstrated in Table 4 and the observed and predicted CH_4_ production (g/d) values are illustrated in Figure 1 and Figure 2. Overall, for the COM database, all the equations had RMSPE, RSR, and CCC values ranging between 12.2–30.0%, 0.34–0.82, and 0.71–0.94, respectively, while for the LAC database, these values ranged from 9.7 to 26.9%, 0.43 to 1.02, and 0.56 to 0.89, respectively. Among the extant models evaluated, the equations of Ellis et al. [18], Mills et al. [20], Ramin and Huhtanen [72], Patra [87], Storlien et al. [89], Moraes et al. [90] revealed systematic biases (SB and MB, *p* < 0.05) with a greater RMSPE ranging from 17.6 to 30.0% for the COM database and 15.2 to 26.9% for the LAC database (Figure 2). The equation of Storlien et al. [89] exhibited a low precision among all the published equations, with RMSPE >26% (higher RSR) and a lower accuracy, as evidenced by CCC values below 0.71 for both databases. The equations of Ellis et al. [18], Patra [87], Moraes et al. [90] underpredicted CH_4_ production on COM and LAC databases at the upper values (Figure 1 and Figure 2), with both systematic biases and greater RMSPE (20.6 and 20.5%, respectively; Figure 1). In contrast, the IPCC [19] and IPCC [86] Eq. 1 models revealed no slope biases on COM (SB = 1.93 and 2.71%, respectively) and LAC (SB = 0.07 and 5.04%, respectively) databases; however, they showed a significant mean bias on both databases (MB > 14%). Moreover, these equations exhibited an overestimation or underestimation of CH_4_ production at the upper values (Figure 1 and Figure 2). In contrast, the Niu et al. [16] equation did slightly better with no mean bias but showed significant slope biases on COM (SB = 56.84%) and LAC (SB = 45.57%) databases. The Niu et al. [16] equation underpredicted CH_4_ production on COM and LAC databases with a maximum bias of 96.3 g/d and 109.1 g/d, but this bias was greater than 39.11 and 39.02 g/d predicted standard errors, respectively. The models of IPCC [19], Yan et al. [88], Hristov et al. [92] had similar RMSPEs of 14.2, 14.5, and 14.4%, respectively, and they also exhibited mean biases ranging from 13.66 to 25.12% (*p* < 0.05) but showed no slope biases (*p* > 0.05). Similarly, on the LAC database, the same models resulted in similar prediction errors and CCC values (RMSPE = 16.6 − 14.0; CCC = 0.82 − 0.83) associated with the mean bias. In contrast, the models of Charmley et al. [17], IPCC [86] were ranked second among all the extant models evaluated on both COM and LAC databases. These models demonstrated a comparable predictive performance and accuracy without any systematic errors (Table 4, Figure 1 and Figure 2).

Among the 15 chosen extant models evaluated with both COM and LAC databases, the equation of Nielsen et al. [91] had the best precision and accuracy (Table 4, Figure 1 and Figure 2) with the lowest RMSPE (<12%) and greater CCC (>0.89) but did not outperform the newly developed models in the present study. Despite the observation that the Nielsen et al. [91] model demonstrated an identical prediction error to our best-performed model evaluated with the LAC database, the CCC analysis showed that our model had a better prediction accuracy (0.94 vs. 0.89). These results suggest that given the available data, there are no benefits of including additional dietary variables such as EE and dNDF, as done in the Nielsen et al. [91] model, compared to our model that incorporates DMI, dietary forage proportion, and a quadratic term of dietary forage proportion. 

These results demonstrate that an increase in model complexity by the inclusion of the dietary components could enhance the ability to forecast CH_4_ production because systematic bias was observed when DMI was the sole predictor, leaving much variability unexplained.

**Table 4:** Published methane production (g/d) equations evaluation using combined (COM; *n* = 61) and lactating cows (LAC; *n* = 48) databases.

		Model Performance ^b^				
Extant Models	Equations ^a^	RMSPE, %	RSR	MB, %	SB, %	CCC
**Combined (COM)**						
Yan et al. [88]	3.23 (0.523) + 0.055 (0.0018) × IE	14.5	0.40	17.08	6.05	0.91
Mills et al. [20]	5.93 (1.60) + 0.92 (0.08) × DMI	17.6	0.48	35.92	13.88	0.86
IPCC [19] Tier 2	(0.065 × IE)/0.05565	14.2	0.39	13.66	1.93	0.92
Ellis et al. [18]	3.23 (1.12) + 0.809 (0.0862) × DMI	20.6	0.56	40.14	23.09	0.80
Hristov et al. [92]	2.54 (4.89) + 19.14 (0.43) × DMI	14.4	0.39	22.60	2.36	0.91
Nielsen et al. [91] ^c^	1.23 × DMI − 1.45 × EE + 0.171 × dNDF	12.2	0.34	4.37	7.60	0.94
Ramin and Huhtanen [72]	20 (12.1) + 35.8 (2.87) × DMI − 0.50 (0.132) × DMI^2^	18.1	0.49	6.87	29.14	0.83
Moraes et al. [90]	−0.163 + 0.051 × IE + 0.038 × NDF	17.6	0.48	40.09	13.24	0.86
Storlien et al. [89]	6.80 + 1.09 × DMI − 0.15 × EE	30.0	0.82	82.05	0.33	0.71
Charmley et al. [17]	38.0 (29.03) + 19.22 (1.40) × DMI	13.2	0.36	8.10	2.49	0.93
Patra [87]	1.29 (0.906) + 0.878 (0.125) × DMI	22.6	0.62	57.44	12.00	0.78
Patra [87]	71.47 (22.14) × (1 − exp (−0.0156 (0.0051) × DMI)	22.0	0.60	52.96	13.19	0.79
Niu et al. [16]	33.2 (13.54) + 13.6 (0.33) × DMI + 2.43 (0.245) × NDF	16.9	0.46	0.00	56.84	0.84
IPCC [86] Eq. 1	(0.057 × IE)/0.05565	15.0	0.41	25.12	2.71	0.91
IPCC [86] Eq. 2	(0.060 × IE)/0.05565	13.1	0.36	4.14	0.38	0.93
**Lactating (LAC)**						
Yan et al. [88]	3.23 (0.523) + 0.055 (0.0018) × IE	13.6	0.58	10.90	8.43	0.83
Mills et al. [20]	5.93 (1.60) + 0.92 (0.08) × DMI	15.2	0.58	23.98	14.21	0.78
IPCC [19] Tier 2	(0.065 × IE)/0.05565	13.8	0.52	16.72	0.07	0.84
Ellis et al. [18]	3.23 (1.12) + 0.809 (0.0862) × DMI	20.5	0.78	51.40	14.62	0.63
Hristov et al. [92]	2.54 (4.89) + 19.14 (0.43) × DMI	14.0	0.53	22.48	4.51	0.83
Nielsen et al. [91] ^c^	1.23 × DMI − 1.45 × EE + 0.171 × dNDF	9.7	0.43	0.31	4.22	0.89
Ramin and Huhtanen [72]	20 (12.1) + 35.8 (2.87) × DMI − 0.50 (0.132) × DMI^2^	17.7	0.63	14.32	41.54	0.64
Moraes et al. [90]	−0.163 + 0.051 × IE + 0.038 × NDF	17.3	0.66	44.76	11.26	0.73
Storlien et al. [89]	6.80 + 1.09 × DMI − 0.15 × EE	26.9	1.02	78.91	1.14	0.56
Charmley et al. [17]	38.0 (29.03) + 19.22 (1.40) × DMI	12.6	0.48	5.16	5.23	0.86
Patra [87]	1.29 (0.906) + 0.878 (0.125) × DMI	22.3	0.85	62.7	8.58	0.61
Patra [87]	71.47 (22.14) × (1 − exp{−0.0156 (0.0051) × DMI	21.7	0.82	57.48	13.00	0.61
Niu et al. [16]	33.2 (13.54) + 13.6 (0.33) × DMI + 2.43 (0.245) × NDF	14.3	0.54	7.64	45.57	0.77
IPCC [86] Eq. 1	(0.057 × IE)/0.05565	14.6	0.55	24.14	5.04	0.81
IPCC [86] Eq. 2	(0.060 × IE)/0.05565	12.7	0.48	3.20	3.10	0.86

^a^ DMI = dry matter intake (kg/d); IE = intake energy (MJ/d); NDF = neutral detergent fiber (% of DM); EE = ether extract (% of DM); dNDF = digestible neutral detergent fiber (% of DM). In parentheses are the standard errors (SE). ^b^ RMSPE = root mean square prediction error expressed as the percentage of the observed mean daily methane production (g/d); RSR = RMSPE-observations standard deviation ratio; MB = mean bias expressed as the percentage of the total mean square prediction error; SB = slope bias expressed as the percentage of the total mean square prediction error; CCC = concordance correlation coefficient. Figure 1 and Figure 2 illustrate the performance of the published models on combined cows (COM) and lactating cows (LAC) data. ^c^ Nielsen et al. [91] model was fitted with 43 and 30 observations on COM and LAC databases, respectively.

#### 3.3.2. Methane Yield (g/kg DMI) Equations

The CH_4_ yield regression equations were developed on COM (Eqs. 1–4) and LAC (Eqs. 5–9) databases, and their performances are presented in Table 5 (Figure S2). Lactation performance variables were also evaluated using the LAC database and resulted in model 9 (diet_lac_M). The methane yield had a negative relationship with dietary CP (Eq. 4), NFC (Eq. 8), and MPY (Eq. 9) and was positively related to NDF (Eqs. 1, and 5), ADF (Eqs. 2, and 6), dietary forage proportion (Eqs. 3, and 7), and MFY (Eq. 9). 

All the models’ predictions show variability among the data. Both NDF_S and ADF_S had a similar prediction performance with RMSPEs of 11.7 and 11.8%, respectively, on the LAC database. However, Eq. 2 (ADF_S) showed a slightly greater RMSPE of 12.1% and a lower CCC of 0.30 compared to Eq. 1 (NDF_S) with prediction errors of 11.5% and a greater CCC of 0.49 with the COM database. For the LAC database, the forage proportion-only predictor (Forage_S; Eq. 7) model showed a better prediction accuracy than NDF_S (Eq. 5) and ADF_S (Eq. 6), with the lowest RSR and greater CCC values (RSR = 0.73, CCC = 0.61), but that was not the case on the COM database (RSR = 0.82, CCC = 0.46). Model variable selection in diet_M (Eq. 4) fitted on COM and LAC databases resulted in different predictor variables. The model developed using the COM database resulted in the parameterization of dietary forage proportion and CP, whereas the LAC database model resulted in dietary forage proportion and NFC. However, all these models also retained the dietary forage proportion variable. Diet_M on COM data showed a slightly greater RMSPE (12.6%) compared to the dietary forage proportion-only predictor model (Forage_S) with an RMSPE of 11.4%, and this model overpredicted CH_4_ yield at the upper end. A similar pattern was found in the LAC database (RMSPE = 10.4%), but this tended to underpredict the CH_4_ yield at the upper end. Overall, among all the models developed on the LAC database, the best model was identified with the variable selection that resulted in dietary forage proportion, MFY, and MPY. This model had the lowest prediction error (RMSPE = 7.4%) and greatest CCC (0.77) with a 99.99% error due to random sources. For COM data, the simple regression models of dietary forage proportion (Forage_S) and neutral detergent fiber (NDF_S) showed a better prediction accuracy on CH_4_ yield compared to other models.

Although the extant models on both databases demonstrated negligible slope biases and had comparable prediction errors (RMSPE > 11.0%; Eqs. 1, 2 and 3; Table 5) and CCC values (ranged from 0.39 to 0.44), Eq. 3 of Niu et al. [16] had a lower prediction accuracy (CCC = 0.25) and greater RSR (0.93). Among all the published models in Table 5, Niu et al. [16] Eq. 1 fitted with an NDF-only predictor was ranked the highest based on the slightly lower RMSPE, RSR, and slightly greater CCC value. 

All newly developed models showed negligible mean and slope biases to extant equations.

## 4. Discussion

The first objective of this study was to investigate the effects of monensin on CH_4_ emissions while also developing predictive enteric CH_4_ emission models; however, with the paper’s inclusion/exclusion criteria for the monensin database, no robust model was developed due to insufficient observations. The third objective was achieved using a database comprised both of lactating and non-lactating cows (i.e., heifers and dry cows), and we did not observe differences in the relationship between CH_4_ emissions (g/d or g/kg of DMI) and input variables when assessed on separate datasets as either lactating or non-lactating cows. As a result, the final statistical data analysis was performed using the combined data. This approach is similar to that of Moe and Tyrrell [96] who developed predictive enteric CH_4_ production models using combined data from lactating and dry cows.

### 4.1. Effects of Monensin on Methane Emissions

Grazing studies that evaluated the effects of monensin on CH_4_ production were eliminated from the database for several reasons. After careful reading of the papers, there was no analysis and statement of the monensin concentration in the diet or what was consumed, thus there was no way to verify the monensin concentration. More mechanistically, there are concerns with the dosing of the monensin in several published studies as monensin was fed once or twice daily in what could be considered bolus dosing due to how grazing cows are supplemented at milking times. This contrasts with cattle consuming total mixed rations (TMR) where the monensin is thoroughly mixed and cattle consume some monensin with every feeding bout. Additionally, the solubility of monensin is approximately 20% [97], so with a once- or twice-a-day intake, the ability to distribute the monensin uniformly in the rumen is likely reduced. This is likely compounded by the liquid passage rate and turnover in grazing cattle consuming high-quality pasture grass. In the grazing study of Dineen et al. [98], the liquid passage rate was approximately 0.21 per hour. This suggests the rumen turnover rate for the liquid fraction in grazing cattle is approximately 5 h, a very rapid turnover compared to TMR-fed cattle where it would be about half that rate. Thus, with no verified monensin intake, a low solubility, non-uniform intake, and rapid rates of liquid passage, the data were not considered in the evaluation of monensin effects on CH_4_ production and might explain why the results from those studies are so varied.

Regardless of the issue encountered related to the limited number of observations in the monensin database, in vivo studies that had measured daily CH_4_ production longer than 21 days following monensin supplementation were used to evaluate the impact of monensin on enteric CH_4_ emissions. Unexpectedly, all the remaining studies (*n* = 6) in the monensin database had supplemented monensin at 24 mg/kg DM of feed. The present study observed a significant reduction in daily enteric CH_4_ production (g/d) of 5.4% and CH_4_ yield (g/kg DMI) of 4.0%, with monensin supplementation at 24 mg/kg DM. These values are within the range reported by the reviews of Beauchemin et al. [27], Beauchemin et al. [99], which showed that monensin at 24 mg/kg can reduce CH_4_ production (g/d) by 4–10% and CH_4_ yield (g/kg DMI) by 3–8% in dairy cows, and this also supports the findings that the monensin effects on methanogenesis are dose-dependent [99]. In addition, NASEM [35] reported a 5% reduction in CH_4_ production with monensin inclusion in the diet. Furthermore, the current study suggests the timing of taking CH_4_ measurements after the initiation of monensin feeding is crucial to obtain clear effects of monensin on methanogenesis in dairy cattle. Thus, the absence of consistent findings in earlier research and the lack of significant CH_4_ reduction in monensin-fed dairy cattle point to insufficient time being given to the ruminal microorganisms to be exposed to monensin. This is supported by the data of Odongo et al. [30], who observed a significant decrease in CH_4_ production after 30 days when evaluating the long-term feeding (i.e., 6 months) of dairy cattle with monensin at a dose of 24 mg/kg DM. This indicates that the timing of the CH_4_ measurement after monensin feeding has been overlooked in recent research.

### 4.2. Key Predictors of Methane Production and Yield

In the present study, the key significant predictors of CH_4_ production (g/d) and CH_4_ yield (g/kg DMI) were identified using different model categories fitted in a linear mixed model procedure. Similar to earlier studies [15,16,17,22,72], DMI was the significant predictor of enteric CH_4_ production in dairy cattle for COM and LAC databases and was positively related to daily enteric CH_4_ production on both databases (*r* = 0.94, *p* < 0.001 and *r* = 0.89, *p* < 0.001 for COM and LAC, respectively). Greater DMI results in more CH_4_ production due to the increased availability of substrates for digestion and related byproducts, i.e., hydrogen ion (H_2_) for rumen microbial fermentation. The slope of DMI corresponding with CH_4_ production in COM and LAC databases ranged from 16.8 to 17.8 and 16.6 to 20.6 g/kg of DMI, respectively. These ranges are greater than the range of 13.0 to 15.3 g/kg DMI reported by Niu et al. [16] for European dairy cows. In a simple model of DMI (DMI_S, Table 3) for the COM database (*R^2^* = 0.87), every additional kg of DMI was associated with an increase of 16.8 g in CH_4_ production, whereas on the lactating database (LAC), it was 18.4 g/kg DMI (*R^2^* = 0.74) on average. This slope for lactating cows (18.4 g/kg DMI) was similar to that reported by Congio et al. [15] but lower than that of Charmley et al. [17] (20.7 g/kg DMI) for the universal equation developed from beef and dairy cattle records (*n* = 1034) fed tropical and temperate forages. These differences could be a result of the differences and variability in dietary composition and forage digestibility. In addition, IE and MEI showed a strong significant positive correlation to CH_4_ production (g/d), and these results are in agreement with other studies [14,18].

It has been estimated that enteric CH_4_ loss accounts for a significant portion of feed energy loss from 2 to 12% [3], and the values from the present analysis fall within these ranges, showing CH_4_ loss as a percentage of IE (Y_m_) ranging from 4.0 to 7.8% with an average of 6.2% (SD = 0.84) and 6.0% (SD = 0.83) for the COM and LAC datasets, respectively (Table 2). However, these values are slightly lower than that reported by the IPCC [19] of 6.5% but are similar to that of IPCC [86] Eq. 2 (6.0%). The [19] slope-only model showed an overprediction of CH_4_ production in the current study with a significant mean bias as assessed according to St-Pierre [83] (−18.9 g/d, *p* = 0.002), and these findings are consistent to those reported by others [16,22,100]. In contrast to our results, Appuhamy et al. [22] observed 5.7% Y_m_ in North American lactating dairy cows. In ruminants, forage preservation methods have been shown to modify CH_4_ production in forage-based diets [13], with a lower CH_4_ loss as a percent of IE with silage-based diets than hay in vitro [101], while others did not observe any differences in vivo [102]. Therefore, slightly lower Y_m_ values in our study might be related to the non-structural carbohydrates associated with feeding silages as the majority of the studies in our database (78%) were fed silages as forage sources or sole-forage sources in the diets than they did dry hay (22%).

The NDF fraction represents the majority of the cell wall content of forage, and the structural polysaccharides hemicellulose and cellulose, with the greater content being cellulose, followed by hemicellulose [103]. This chemical fraction varies among forage species [103], and fiber digestibility is crucial for enteric CH_4_ production. A diet high in NDF results in a longer ruminal retention time, which encourages an increase in the availability of the methanogenic substrate, H_2_, from the acetate and butyrate production for CH_4_ synthesis [3]. This could explain the positive relationships found between CH_4_ production and dietary forage proportion and the CH_4_ yield and NDF or ADF content found in the regression analysis in the present study. For the COM and LAC databases, the Pearson correlation analysis between dietary forage proportion and CH_4_ production showed a tendency not to be significantly related (results not shown); however, the dietary forage proportion variable showed a better CH_4_ emission prediction accuracy in the regression analysis. A nonlinear relationship between dietary forage proportion and CH_4_ production with a dietary forage proportion range of 40–100% was observed in this study, and the results are similar to that suggested by Lovett et al. [104], Benchaar et al. [105]. We observed a decline in CH_4_ production at higher dietary forage proportion inclusion levels, and this depressed CH_4_ production observed at a higher forage might be related to the greater digestibility of the forage sources fed.

In contrast to greater CH_4_ emissions from the fibrous CHO, less CH_4_ emissions in dairy cattle have been observed with higher DMI of NFC. A negative effect of NFC on CH_4_ yield was observed in the current study. An increase in the prediction error from 9.9 to 10.4% in the CH_4_ yield model when adding NFC along with forage proportion was observed in our analysis, but this model was outperformed by the simple model of forage proportion. This agrees with the observations of Ramin and Huhtanen [72] who observed a minor contribution of dietary carbohydrates (NFC/NDF and NDF) to the CH_4_ prediction accuracy. Moreover, the NFC measurement could be unreliable to use to predict CH_4_ emissions because it is calculated using the other dietary component variables.

Contrary to other studies [16,18], no obvious relationship was observed between CH_4_ production and NDF on both combined and lactating cow databases (*r* = 0.04 and 0.15, respectively), but CH_4_ production was positively correlated with NDFd for LAC as expected. This result shows that NDFd can be a better predictor of CH_4_ production in lactating dairy cows. Furthermore, CH_4_ yield (g/kg DMI) was positively related to NDF and ADF on combined and lactating cows’ databases, and this is similar to Hammond et al. [106]. Consistent with the recent studies [107,108], less CH_4_ production was observed in our study with the increase in the H:C ratio (COM: *r* = −0.50, *p* < 0.001; LAC: *r* = −0.59, *p* < 0.001). Hemicellulose is highly digestible and it hydrolyzes at a faster rate than cellulose, yielding less CH_4_ production if available to the bacteria. However, in grasses, one other observation is that the concept of lignification includes the para-coumaric and ferulic ester and ether linkages between lignin and hemicellulose [109], and these linkages can impact the rate and extent of the digestion of hemicellulose and cellulose [110]. This supports the increase in enteric daily CH_4_ production with an increase in the cellulose concentration observed in our study, and these results are similar to that of Ma et al. [107]. Moe and Tyrrell [96] reported a 37% reduction in CH_4_ production with an increase in digested hemicellulose compared to the digestion of cellulose.

Similar to other studies, positive model coefficients were observed between CH_4_ emissions, MY, and MFY in lactating cows. The positive association between CH_4_ production and MY or ECM is the result of the dilution effect of an increase in DMI, as observed by the positive relationship between DMI and MY (*r* = 0.94, *p* < 0.001) or DMI and ECM (*r* = 0.91, *p* < 0.001). In the current study, MPY was associated with a decrease in CH_4_ emissions, and this agrees with Velarde-Guillén et al. [100]. This is likely related to a greater propionate production yielding more glucose, microbial protein, and mammary protein synthesis signaling. On the contrary, the fermentation of NDF in dairy cattle diets encourages the production of acetate, resulting in mammary fatty acid synthesis and elongation [111]. This observation supports the positive relationship found between CH_4_ production or CH_4_ yield (g/kg of DMI) and MFY.

### 4.3. Newly Developed and Extant Model Performance for Methane Production (g/d)

To account for the accuracy of the reported CH_4_ production in studies, the models developed were weighted by the number of animals in the study, and if the regression slope bias was found to be significant at *p* < 0.05, then the magnitude of bias was quantified following the recommendation of St-Pierre [83]. The models developed from the COM database included animal input variables such as DMI/IE/MEI and dietary components only, while the LAC database also included the lactation performance variables such as ECM, MY, and milk composition. The models developed in the current study indicated that DMI, as the only predictor on the COM and LAC databases, had a similar predictive accuracy in terms of CH_4_ production with RMSPEs of 14.7 and 14.1 %, accounting for 87 and 74%, respectively, in CH_4_ production. Previous studies developed empirical prediction CH_4_ production models in dairy cattle, though their findings contradict our study, suggesting the simple models of DMI or IE can be sufficient to predict CH_4_ production in dairy cattle with the lowest RMSPE values [15,18]. For example, the simple regression DMI models have proven to better predict enteric CH_4_ production in beef cattle [21], dairy cattle [16], and both dairy and beef cattle [18]. For the COM database in the current analysis, the DMI-only model (DMI_S) exhibited no mean bias but a significant slope error of 17.6% (*p* < 0.001; Figure 1) with overprediction at the lower end and underprediction at the upper end. This model had a maximum bias of 45.8 g/d at the upper end; however, its biases are smaller than the standard error of prediction (46.9 g/d). A slight underprediction with this equation was observed on the LAC database, but with the absence of mean and slope biases found (Figure 2), and this supports the results of Congio et al. [15].

For the COM database, all the models were developed with the DMI because models without the use of DMI showed a poor accuracy and precision (results not shown, no significant dietary component variables); therefore, this could be an indication that DMI cannot be assumed to be constant across treatment means or studies and it has been stated that it represents both animal and plant characteristics which affect rumen fermentation [17]. This is not surprising because DMI explained 87% of the variation in enteric CH_4_ production in the present study. Similarly, Niu et al. [16] observed the poorest prediction accuracy of the model (Eq. 24) when DMI was taken out of the models as supported by an increase in the RMSPE of 15.8% on the EU database compared to the model that used DMI and NDF (RMSPE = 14.7%).

The best CH_4_ production prediction models for both COM and LAC databases, ranked by the lowest RSMPE (10.7 and 9.1%, respectively) and RSR values (0.29 and 0.34, respectively) required DMI, dietary forage proportion, and a quadratic term of dietary forage proportion (DMI_For_nl). The observed nonlinear relationship between dietary forage proportion and CH_4_ production demonstrated a reduction in CH_4_ production at higher dietary forage proportion inclusion, and this might be explained by the fact that our databases were not constrained to any forage-inclusion levels, and ranged from 40 to 100%, and with greater forage intake, forage digestibility usually increases.

In contrast to our findings, the study of Ellis et al. [18] did not find an improvement in the model performance with dietary forage proportion and CH_4_ production in the combined database of beef and dairy cattle, but other previous studies have [104,105], including the present study. The current analysis showed the existence of a curvilinear relationship between dietary forage proportion and CH_4_ production at higher dietary forage proportion inclusion. Patel et al. [66] revealed lower enteric CH_4_ production with feeding grass silage at greater than 50% of total DMI with NDF values less than 400 g/kg DM in the diets. This could explain the depressed enteric CH_4_ production with an increase in dietary forage proportion in the present study, with the lower average dietary NDF values of 35.3 and 34.7% of DM for COM and LAC databases, respectively.

When dietary forage proportion was excluded in the model development process, the second-ranked model based on the lowest RMSPE on the COM database, resulting in DMI, Lig.%NDF, and H:C ratio (noForage_diet_M: RMSPE = 10.9%). This model was comparable to the best-selected model that required DMI, dietary forage proportion, and the quadratic response of dietary forage proportion to CH_4_ production (DMI_For_nl: RMSPE = 10.7%; Table 3). As a result, it can be challenging to identify the most accurate and precise model; however, based on the availability of input variables on the farm, dietary forage proportion (% DM) and DMI might be sufficient for accurate predictions.

The use of the lactation performance variables has been reported to improve CH_4_ emissions predictions [16]. Even though the model that included the DMI, MPY, and MFY outperformed other models developed using the LAC database in terms of the lowest RMSPE and RSR values (RMSPE = 9.8%; RSR = 0.40), it did not outperform models with input variables selected using the combined data and evaluated on the LAC database, i.e., models that used DMI, dietary forage proportion, and the quadratic response of dietary forage proportion (DMI_For_nl: RMSPE = 9.1%, RSR = 0.34; Table 3) or DMI, dietary lignin (% NDF), and dietary forage proportion (RMSPE = 9.3%; RSR = 0.35). Therefore, the use of dietary components can be sufficient to use for enteric CH_4_ prediction in lactating cows as well.

In the current study, the equation of Nielsen et al. [91] outperformed all the published models on both COM and LAC databases (RSR = 0.34 and 0.43; RMSPE = 12.2 and 9.7%; CCC = 0.94 and 0.89, respectively) with error due to random sources of greater than 88%, and the least prediction accuracy was observed with the Storlien et al. [89] equation. These findings are consistent with those reported by Appuhamy et al. [22], who found the best CH_4_ emissions predictions for North American cows using Nielsen et al. [91] equation. It is worth noting that this model requires predictor variables such as DMI, dietary fatty acids (FAs), and dNDF contents, of which we did not have the FAs in our database. As a solution, we initially estimated the FAs using the equation of Giger-Reverdin et al. [112] as cited by Appuhamy et al. [22]. However, the model’s prediction performance was poor (results not shown). We then replaced the FAs with the EE from our database, the model exhibited the best performance among all the chosen published models in the present study. These results suggest that the use of an easily accessible dietary variable such as EE may be used instead of FAs.

The second-ranked updated published equation of IPCC [86] (Eq. 2, Table 4) demonstrated no systematic biases with a better prediction performance of CH_4_ production, and this agrees with a recent study that reported that the equation adequately predicted CH_4_ emission from lactating cows who were fed Mediterranean diets [113]. Despite some slight underestimation of CH_4_ emissions with the IPCC [86] at the upper values, our study demonstrated that the refined factors in this model had improved CH_4_ production predictions compared to the IPCC [19] model, which tended to overpredict at higher values. A better CH_4_ prediction performance observed with the IPCC [86] given our databases could be explained by the identical average Y_m_ values (COM: 6.2 ± 0.84%; LAC: 6.0 ± 0.83%). Furthermore, the model of Charmley et al. [17], which was developed only based on the CH_4_ measurements from Australian cows, exhibited a better prediction and was comparable to the IPCC [86]. In agreement with the study of van Lingen et al. [21], the equation of Charmley et al. [17] also outperformed the simple regression of DMI developed in the present study with slightly lower RMSPE and RSR values of 13.2% and 0.36, respectively. The discrepancy in performance is likely caused by ranges in stages of the lactation and maturity of cattle in our databases linked to ranges in DMI. An improved CH_4_ prediction performance of the equation of Charmley et al. [17] given our database could be attributed to the fact that their database for model development included Australian studies that fed high-forage-based diets (>70%), which is consistent with the database employed in the present investigation.

Overall, the analysis demonstrated a better CH_4_ prediction accuracy with extreme values of dietary forage proportion (40–100%) along with DMI, revealing a nonlinear relationship with CH_4_ production, supporting the results of Mills et al. [20] who suggested an improvement in the prediction at extreme values under the practical application. Even though we were unable to develop a robust CH_4_ production predictive model from the monensin-only database, our preliminary analysis indicated that the same predictor variables, such as Lig.%NDF, cellulose, and the H:C ratio, have shown to be the key predictors of CH_4_ production, which is similar to the current analysis.

### 4.4. Methane Yield (g/kg DMI) Model Performance 

In contrast to the CH_4_ production model findings in the present study, the simple regression of NDF was significant (g CH_4_/kg of DMI = 16.99 (1.41) + 0.11 (0.037) × NDF (% of DM); RMSPE = 13.1%; R^2^ = 0.09) from the COM database (Table 5). However, this model is outperformed by the dietary forage proportion-only predictor model with an RMSPE of 11.5% from the COM database, and our results corroborate earlier work [114] in sheep.

For the LAC database, an improvement in the CH_4_ yield prediction was observed with the increase in model complexity (diet_lac_M), including dietary forage proportion (% of DM), milk fat yield, and milk protein yield variables (RMSPE = 7.4%; CCC = 0.77). Consistent with our study, Niu et al. [16] observed a better CH_4_ yield prediction with milk composition with an RMSPE value of 16.1%. The published equation by Niu et al. [16] using a NDF-only predictor was ranked high compared to other extant models, with the lowest RMSPE and highest CCC on both databases.

In the current study, we developed robust models for an enteric CH_4_ production and yield for both lactating and non-lactating cows, with easily accessible input variables. Dietary laboratory analysis and CH_4_ emission measurement techniques can be very expensive; therefore, the use of these empirical CH_4_ prediction equations developed in the present study can be used with easily available inputs.

## 5. Conclusions

The databases of COM and LAC were compiled to develop the models for enteric CH_4_ production from dairy cattle. This study revealed that DMI is the primary predictor of CH_4_ production in dairy cattle; however, an improvement in the CH_4_ production prediction accuracy was found with an increase in the model complexity by the inclusion of the dietary components. The current best-developed models have shown a better CH_4_ prediction performance than the selected extant models. Among all the published models, the Nielsen et al. [91] model recommended by NASEM [35] improved the CH_4_ emission predictions evaluated on both databases. The present study demonstrated that an enteric CH_4_ production and yield can be predicted by factors such as DMI, dietary forage proportion, and lactation performance variables. These newly developed CH_4_ production equations can be used to estimate CH_4_ emissions with easily accessible input variables.

Furthermore, monensin supplementation reduced enteric CH_4_ production and yield, and this study has shown that the timing of CH_4_ measurements following monensin supplementation is crucial. Thus, long-term in vivo studies with fully adapted rumen microbial populations are needed.

## Figures and Tables

**Table 1:** Summary of the database used for the model development.

Author	CH_4_ Collection Technique	N	CH_4_, (g/d) Mean (*SD*)	CH_4_/DMI, (g/kg) Mean (*SD*)	DMI (kg) Mean (*SD*)
[58]	Chamber	2	275.5 (74.77)	19.8 (3.40)	13.8 (1.41)
[59]	Chamber	2	194.4 (6.92)	14.4 (1.17)	13.6 (0.62)
[60]	Chamber	4	248.1 (27.37)	17.9 (1.99)	13.9 (0.63)
[61]	Chamber	7	223.2 (82.35)	20.7 (2.05)	11.2 (5.15)
[62]	Chamber	6	249.7 (100.31)	19.2 (2.88)	13.5 (6.37)
[63]	Chamber	4	165.6 (11.67)	24.2 (2.72)	6.9 (0.42)
[64]	Chamber	4	386.8 (51.85)	23.0 (2.31)	17.1 (3.72)
[30]	Hood	2	443.7 (21.21)	22.9 (0.57)	19.4 (0.42)
[65]	Chamber	4	471.8 (21.82)	23.7 (1.19)	19.7 (0.67)
[66]	SF_6_	3	301.0 (19.52)	19.8 (1.08)	15.5 (0.35)
[67]	Chamber	3	452.3 (26.73)	19.8 (1.63)	22.5 (0.70)
[53]	Hood	4	583.8 (37.71)	20.6 (0.77)	28.3 (1.01)
[32]	SF_6_	2	354.9 (39.95)	17.5 (2.33)	20.3 (0.57)
[68]	Chamber	4	415.3 (19.19)	21.9 (0.44)	19.0 (0.49)
[69]	Chamber	4	402.8 (12.34)	24.0 (1.37)	16.8 (0.54)
[31]	SF_6_	2	486.0 (14.14)	21.9 (0.85)	22.1 (0.42)
[9]	SF_6_	2	475.0 (18.38)	20.6 (0.42)	23.6 (0.35)
[8]	SF_6_	2	328.4 (64.35)	18.8 (2.74)	17.5 (0.80)

Chamber = respiratory chamber; SF_6_ = sulfur hexafluoride tracer technique; N = number of treatments means per study; *SD* = standard deviation; CH_4_ = enteric methane; DMI = dry matter intake.

**Table 2:** Summary descriptive statistics of the dataset used to develop the proposed methane emission models.

	Combined Database (*n* = 61)				Lactating Database (*n* = 48)			
Input Variable ^a^	Mean	*SD* ^b^	Min ^b^	Max ^b^	Mean	*SD* ^b^	Min ^b^	Max ^b^
Animal variables								
DMI, kg/d	16.7	5.83	6.3	29.2	18.9	4.16	12.8	29.2
IE, MJ/d	312.8	107.83	113.7	546.0	353.3	75.94	243.3	546.0
MEI, MJ/d	117.5	67.35	48.6	313.8	200.5	49.41	129.3	313.8
Dietary nutrient content (% of DM), unless stated otherwise								
CP	15.7	3.18	5.1	22.5	16.2	2.41	6.7	20.2
NDF	35.3	8.01	24.7	69.6	34.7	6.9	24.7	56.4
ADF	21.5	5.59	13.7	42.9	21.2	4.67	13.7	35.2
Lignin, %NDF	11.6	4.05	3.3	18.5	11.2	4.18	3.3	18.5
Lignin	3.9	1.42	1.4	9.2	3.7	1.18	1.4	5.8
EE	3.4	1.06	1.8	7.0	3.4	1.12	1.8	7.0
Ash	6.7	1.51	4.1	11.5	6.7	1.40	4.1	9.9
Hemicellulose	13.8	4.23	5.5	26.7	13.4	4.1	5.5	24.4
Cellulose	17.6	5.2	10.3	33.7	17.6	4.67	10.3	30.1
H:C ratio	0.83	0.26	0.18	1.47	0.80	0.25	0.18	1.47
NFC ^c^	39.0	8.73	18.2	52.9	39.0	8.11	19.3	52.9
Forage, %	57.1	17.66	40.0	100.0	57.3	15.99	40.0	100.0
Performance variables								
MY, kg/d	-	-	-	-	25.6	8.47	10.0	46.3
MFY, g/d	-	-	-	-	1003.9	294.98	490.0	1780.0
MPY, g/d	-	-	-	-	845.4	244.54	370	1430.0
ECM, kg/d^d^	-	-	-	-	26.6	7.35	12.4	44.8
Nutrient digestibility, %								
NDFd	48.5	18.15	12.1	76.9	47.7	19.84	12.1	76.9
CH_4_ emissions								
CH_4_, g/d	346.4	127.13	147.4	631.2	389.7	102.68	189.5	632.2
CH_4_/DMI, g/kg	20.8	2.86	13.5	27.0	20.7	2.64	15.2	25.9
CH_4_/MY, g/kg	-	-	-	-	16.1	2.83	11.4	23.3
CH_4_/ECM, g/kg	-	-	-	-	15.0	1.97	11.3	19.3
Y_m_ (CH_4_/IE), %	6.2	0.84	4.0	7.8	6.0	0.83	4.00	7.6

^a^ CH_4_ = methane; DMI = dry matter intake; IE= dietary intake energy; MEI = metabolizable energy intake; CP = dietary crude protein; NDF = neutral detergent fiber, ADF = acid detergent fiber; Lig.%NDF = lignin as a percent of neutral detergent fiber; EE = ether extract; H:C ratio = hemicellulose to cellulose ratio; NFC = non-fiber carbohydrates; MY = milk yield (kg/d); MFY = milk fat yield (g/d); MPY = milk protein yield (g/d); ECM = energy-corrected milk; NDFd = dietary neutral detergent fiber digestibility (%); Y_m_ = methane conversion factor representing total energy (IE) loss as methane gas. ^b^ *SD* = standard deviation; Min = minimum; Max = maximum. ^c^ NFC (%) = 100 − (NDF + CP + EE + Ash). ^d^ ECM (kg/d) = 12.95 × milk fat yield (kg/d) + 7.65 × milk protein yield (kg/d) + 0.327 × milk yield (kg/d) Tyrrell and Reid [70].

**Table 3:** Methane production (g/d) developed prediction equations and model performance evaluation using combined (COM) and lactating cows (LAC) databases.

		Model Performance ^d^					
Categories ^a^	Prediction Equation ^b^	*n* ^c^	RMSPE, %	RSR	MB, %	SB, %	CCC
**Combined (COM)**							
(1) DMI_S	65.57 (50.53) + 16.77 (0.99) × DMI	61	14.7	0.40	0.07	17.57	0.90
(2) IE_S	66.96 (21.07) + 0.890 (0.054) × IE	61	15.1	0.41	0.05	18.61	0.89
(3) MEI_S	65.82 (24.91) + 1.55 (0.11) × MEI	61	17.8	0.49	0.99	2.44	0.86
(4) DMI_For_M	9.00 (28.39) + 17.62 (0.99) × DMI + 0.73 (0.31) × forage	61	12.8	0.35	0.40	14.44	0.93
(5) DMI_For_nl	−73.74 (27.95) + 17.08 (0.88) × DMI + 2.64 (0.47) × forage − 0.06 (0.01) × forage^2 (centered)^	61	10.7	0.29	1.92	1.75	0.95
(6) DMI_diet_M	45.84 (31.20) + 0.85 (0.29) × forage + 17.84 (0.93) × DMI − 4.26 (1.55) × Lig.%NDF	61	11.5	0.31	0.89	6.40	0.94
(7) noForage_diet_M	169.25 (37.16) + 17.04 (0.94) × DMI − 5.88 (1.73) × Lig.%NDF − 52.19 (19.93) × H:C ratio	61	10.9	0.30	1.04	5.12	0.95
**Lactating cows (LAC)**							
(8) DMI_S	37.67 (42.54) + 18.38 (2.16) × DMI	48	14.1	0.53	1.69	8.22	0.81
(9) IE_S	39.88 (44.03) + 0.98 (0.12) × IE	48	14.5	0.55	1.57	9.64	0.79
(10) MEI_S	128.81 (44.77) + 1.26 (0.21) × MEI	48	18.3	0.69	2.13	6.09	0.63
(11) DMI_For_M	−129.56 (42.51) + 22.63 (1.73) × DMI + 1.56 (0.33) × forage	48	9.5	0.36	0.18	0.00	0.93
(12) DMI_For_nl	−128.95 (37.06) + 19.78 (1.84) × DMI + 2.73 (0.49) × forage − 0.05 (0.02) × forage^2 (centered)^	48	9.1	0.34	1.64	0.00	0.94
(13) DMI_diet_M	−90.73 (56.97) + 1.44 (0.35) × forage + 22.16 (1.77) × DMI − 2.07 (2.00) × Lig.%NDF	48	9.3	0.35	0.43	0.00	0.93
(14) noForage_diet_M	149.04 (53.40) + 18.61 (1.86) × DMI − 5.55 (2.12) × Lig.%NDF − 65.50 (24.37) × H:C ratio	48	10.0	0.38	1.27	1.96	0.92
(15) MY_S	201.58 (33.18) + 7.51 (1.16) × MY	46	16.0	0.66	1.47	3.63	0.68
(16) ECM_S	186.96 (32.43) + 7.86 (1.11) × ECM	46	15.7	0.65	0.59	0.59	0.68
(17) DMI_NDF_M	−93.23 (58.93) + 20.63 (2.02) × DMI + 2.60 (1.05) × NDF	48	11.1	0.42	1.13	7.04	0.89
(18) DMI_ADF_M	−62.91 (51.62) + 20.51(2.01) × DMI + 2.91 (1.29) × ADF	48	11.2	0.42	0.90	3.01	0.89
(19) DMI_diet_lac_M	34.52 (36.51) + 16.64 (3.84) × DMI + 0.225 (0.050) × MFY − 0.214 (0.079) × MPY	46	9.8	0.40	0.08	6.64	0.90
(20) noDMI_diet_lac_M	62.74 (40.32) + 0.162 (0.052) × MFY − 15.52 (6.02) × EE + 4.71 (2.17) × MY + 5.95 (1.68) × cellulose	46	10.1	0.42	0.00	0.37	0.90
(21) NDFd_diet_lac_M	−60.54 (50.81) + 20.13 (2.74) × DMI + 1.35 (0.44) × NDFd	30	11.2	0.49	0.00	3.29	0.87

^a^ Developed model categories: simple models are DMI only (DMI_S); IE_only (IE_S); MEI only (MEI_S); MY only (MY_S); ECM only (ECM_S) and multiple linear models are DMI and dietary forage proportion (DMI_For_M); DMI, dietary forage proportion and quadratic term of dietary forage proportion (DMI_For_nl); DMI and dietary composition variables (DMI_diet_M) and no dietary forage proportion variable but included DMI and dietary nutrient composition variables (noForage_diet_M), DMI and NDF (DMI_NDF_M); DMI and ADF (DMI_ADF_M); DMI, dietary composition and lactation performance variables (DMI_diet_lac_M); no DMI but included dietary and lactation performance variables (noDMI_diet_lac_M) and all variables and NDFd (NDFd_diet_lac_M). ^b^ DMI = dry matter intake (kg/d); IE = intake energy (MJ/d); MEI = metabolizable energy intake (MJ/d); Lig.%NDF = lignin as a percentage of neutral detergent fiber (NDF); MY = milk yield (kg/d); ECM = energy-corrected milk (kg/d); NDF = neutral detergent fiber (%DM); ADF = acid detergent fiber (%DM); MFY = milk fat yield (g/d); MPY = milk protein yield (g/d); NDFd = neutral detergent fiber digestibility (%). In parentheses are the standard errors (SE). ^c^ *n* = is the number of observations in the combined (COM) and lactating data (LAC) used to develop the models. ^d^ RMSPE = root mean square prediction error expressed as the percentage of the observed mean daily methane production (g/d); RSR = RMSPE-observations standard deviation ratio; MB = mean bias expressed as the percentage of the total mean square prediction error; SB = slope bias expressed as the percentage of the total mean square prediction error; CCC = concordance correlation coefficient. Figure 1 and Figure 2 illustrate the performance of these newly developed models.

**Table 5:** Methane yield (g/kg DMI) prediction equations and literature published, and model performance evaluation for combined (COM) and lactating cows (LAC) databases.

		Model Performance ^d^					
Categories ^a^	Prediction Equation ^b^	*n* ^c^	RMSPE, %	RSR	MB, %	SB, %	CCC
**Combined (COM)**							
(1) NDF_S	16.99 (1.41) + 0.11 (0.037) X NDF	60	11.5	0.83	0.00	0.80	0.49
(2) ADF_S	18.49 (1.24) + 0.10 (0.052) × ADF	60	12.1	0.88	0.11	3.06	0.30
(3) Forage_S	16.21 (1.31) + 0.08 (0.021) × forage	60	11.4	0.82	0.23	0.38	0.46
(4) diet_M	21.13 (0.84) + 0.09 (0.02) × forage − 0.35 (0.100) × CP	60	12.6	0.92	0.10	5.58	0.41
Niu et al. [16] Eq.1	13.8 (0.63) + 0.185 (0.0133) × NDF	60	11.5	0.83	6.21	4.58	0.43
Niu et al. [16] Eq. 2	15.4 (0.76) − 0.354 (0.0756) × EE + 0.173 (0.0145) × NDF	60	11.9	0.86	6.26	1.83	0.39
**Lactating cows (LAC)**							
(5) NDF_S	12.24 (1.88) + 0.24 (0.05) × NDF	48	11.7	0.86	0.88	1.79	0.36
(6) ADF_S	14.37 (1.47) + 0.29 (0.07) × ADF	48	11.8	0.87	0.62	2.08	0.33
(7) Forage_S	14.75 (1.00) + 0.10 (0.02) × forage	48	9.9	0.73	0.39	1.28	0.61
(8) diet_M	19.87 (2.38) + 0.08 (0.02) × forage − 0.11 (0.04) × NFC	48	10.4	0.76	0.42	0.01	0.58
(9) diet_lac_M	15.05 (1.52) + 0.08(0.02) × forage + 0.0086 (0.0024) × MFY − 0.0089 (0.0030) × MPY	46	7.4	0.61	0.01	0.00	0.77
Niu et al. [16] Eq. 1	13.8 (0.63) + 0.185 (0.0133) × NDF	48	11.1	0.82	1.90	3.01	0.44
Niu et al. [16] Eq. 2	15.4 (0.76) − 0.354 (0.0756) × EE + 0.173 (0.0145) × NDF	48	11.7	0.86	1.95	0.33	0.39
Niu et al. [16] Eq. 3	21.1 (0.77) + 0.105 (0.0081) × ECM + 1.30 (0.077) × MFP − 0.952 (0.1667) × MPP	46	11.4	0.93	3.86	0.07	0.25

^a^ Developed methane yield (g/kg DMI) model categories: simple models are NDF only (NDF_S); ADF only (ADF_S); dietary forage proportion only (Forage_S), and multiple linear mixed models categories are dietary composition only (diet_M); dietary composition and lactation performance variables (diet_lac_M). ^b^ NDF = neutral detergent fiber (%DM); ADF = acid detergent fiber (%DM); forage = dietary forage proportion (%DM); EE = ether extract (%DM); NFC = non-fiber carbohydrates; MFY = milk fat yield (g/d); MPY = milk protein yield (g/d); MFP = milk fat percent (%); MPP = milk protein percent (%). In parentheses are the standard errors (SE). ^c^ *n* = is the number of observations in the combined (COM: lactating and non-lactating) and lactating data (LAC) used to develop the models. ^d^ RMSPE = root mean square prediction error expressed as the percentage of the observed mean daily methane production (g/d); RSR = RMSPE-observations standard deviation ratio; MB = mean bias expressed as the percentage of the total mean square prediction error; SB = slope bias expressed as the percentage of the total mean square prediction error; CCC = concordance correlation coefficient.

## Data Availability

No data were deposited in the formal repository. Data can be obtained upon request.

## Supplementary Materials

The following supporting information can be downloaded at: https://www.mdpi.com/article/10.3390/ani13081392/s1, Figure S1: Flowchart illustrating the data searching, screening, and selection process used in the current study analysis. Figure S2: Plots of observed vs. predicted methane (CH_4_) yield (g/kg DMI) (illustrated by circles), and residuals (diamond: observed—predicted values) vs. predicted methane yield (g/kg DMI) (illustrated by diamond shapes) generated from the combined (COM) (*n* = 60) and lactating cows (LAC) databases (*n* = 48) from different model categories and extant equations presented in accordance with Table 5. The references interpretations are stated in Table 5. The solid blue lines indicate the relationship between predicted and observed methane production and predicted values and the residuals. The solid black lines represent the line of unity, where y = x (1:1).
