# Supplementary material for: Enteric Methane Emissions Prediction in Dairy Cattle and Effects of Monensin on Methane Emissions: A Meta-Analysis
Source: Animals (Basel). 2023 Apr 18;13(8):1392. doi: 10.3390/ani13081392 (PMC10135289; doi:10.3390/ani13081392)
Supplement: Supplementary file 1 [file animals-13-01392-s001.zip › Figure S1. Flowchart.pdf]

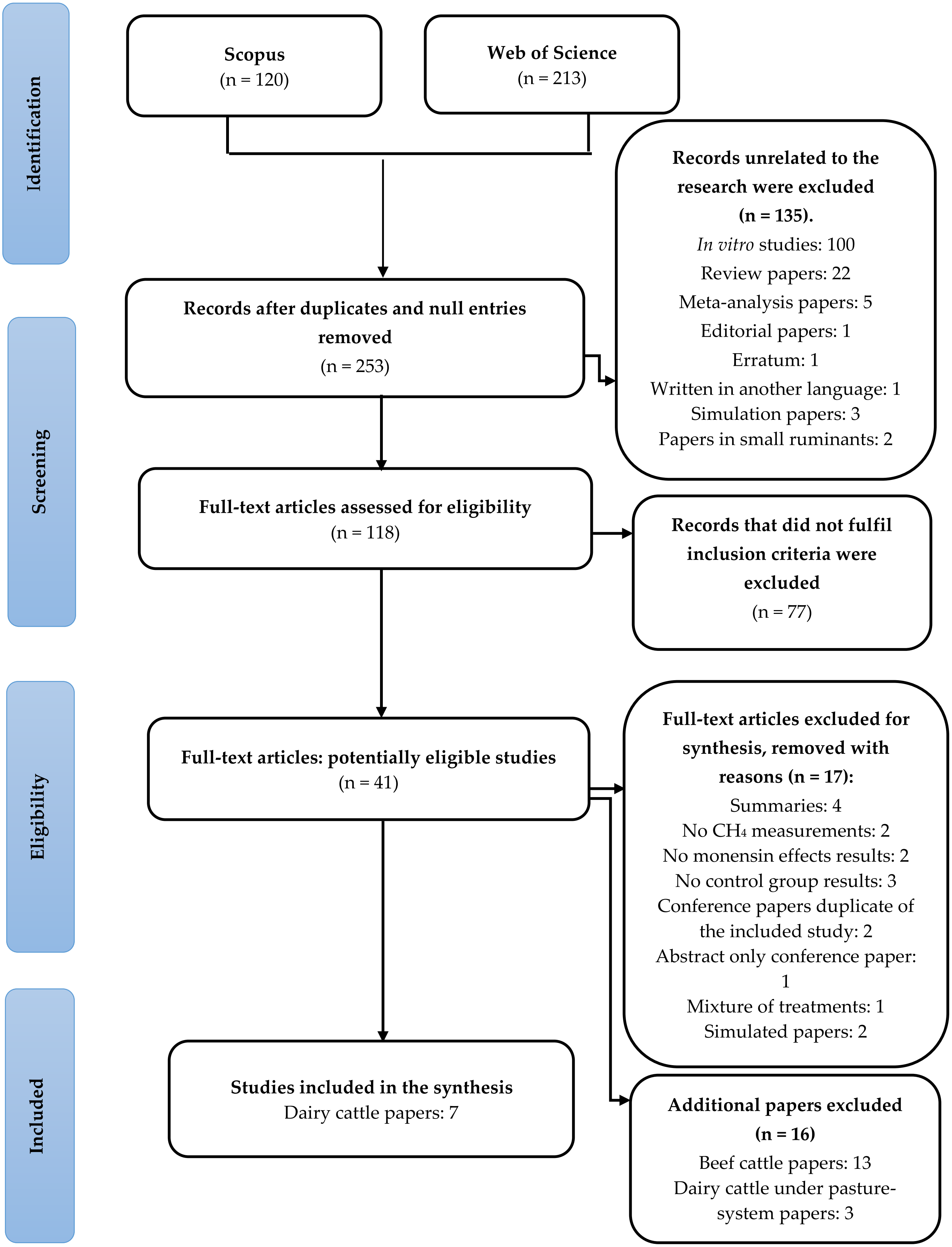
Identification
Screening
Eligibility
Included
Scopus
(n = 120)
Web of Science
(n = 213)
Records unrelated to the research were excluded (n = 135).
In vitro studies: 100
Review papers: 22
Meta-analysis papers: 5
Editorial papers: 1
Erratum: 1
Written in another language: 1
Simulation papers: 3
Papers in small ruminants: 2
Records after duplicates and null entries removed
(n = 253)
Full-text articles assessed for eligibility
(n = 118)
Records that did not fulfil inclusion criteria were excluded
(n = 77)
Full-text articles: potentially eligible studies
(n = 41)
Full-text articles excluded for synthesis, removed with reasons (n = 17):
Summaries: 4
No CH4 measurements: 2
No monensin effects results: 2
No control group results: 3
Conference papers duplicate of the included study: 2
Abstract only conference paper: 1
Mixture of treatments: 1
Simulated papers: 2
Studies included in the synthesis
Dairy cattle papers: 7
Additional papers excluded
(n = 16)
Beef cattle papers: 13
Dairy cattle under pasture-system papers: 3

**Figure S1.** Flowchart illustrating the data searching, screening, and selection process used in the current study analysis.
